# Enhanced Enzymatic Sugar Recovery of Dilute-Acid-Pretreated Corn Stover by Sodium Carbonate Deacetylation

**DOI:** 10.3390/bioengineering10101197

**Published:** 2023-10-14

**Authors:** Weng Fu, Shengbo Wu, Chun Wang, Suchithra Thangalazhy-Gopakumar, Urvi Kothari, Suan Shi, Lujia Han

**Affiliations:** 1 Engineering Laboratory for Agro Biomass Recycling & Valorizing, College of Engineering, China Agricultural University, Beijing 100083, China; fuweng@cau.edu.cn (W.F.); wushengbo_edu@163.com (S.W.); s20213071304@cau.edu.cn (C.W.); hanlj@cau.edu.cn (L.H.); 2 Department of Chemical and Environmental Engineering, University of Nottingham Malaysia Campus, Jalan Broga, Semenyih 43500, Selanggor, Malaysia; suchithra.thangalazhy@nottingham.edu.my; 3 Department of Chemical Engineering, Auburn University, Auburn, AL 36849, USA; urvidk@gmail.com

**Keywords:** deacetylation, sodium carbonate, two-stage pretreatment, enzymatic hydrolysis, acetic acid

## Abstract

The prehydrolysate from dilute acid pretreatment of lignocellulosic feedstocks often contains inhibitory compounds that can seriously inhibit the subsequent enzymatic and fermentation processes. Acetic acid is one of the most representative toxic compounds. In this research, alkaline deacetylation of corn stover was carried out using sodium carbonate under mild conditions to selectively remove the acetyl groups of the biomass and reduce the toxicity of the prehydrolysate. The deacetylation process was optimized by adjusting factors such as temperature, treatment time, and sodium carbonate concentration. Sodium carbonate solutions (2~6 wt%) at 30~50 °C were used for the deacetylation step, followed by dilute acid pretreatment with 1.5% H_2_SO_4_ at 121 °C. Results showed that the acetyl content of the treated corn stover could be reduced up to 87%, while the hemicellulose loss remained low. The optimal deacetylation condition was found to be 40 °C, 6 h, and 4 wt% Na_2_CO_3_, resulting in a removal of 80.55% of the acetyl group in corn stover and a hemicellulose loss of 4.09%. The acetic acid concentration in the acid prehydrolysate decreased from 1.38 to 0.34 g/L. The enzymatic hydrolysis of solid corn stover and the whole slurry after pretreatment increased by 17% and 16%, respectively.

## 1. Introduction

Lignocellulosic biomass is the most abundant renewable feedstock in the world and can be converted into various value-added products, such as biofuels. Crop straws, such as corn stover, are the residual materials left over after crops have been harvested, and they are one of the most representative lignocellulosic biomasses. The production of crop straws in China ranks as the number one in the world [1]. These crop residues are often disposed of through burning or landfilling. These disposal methods could cause air and water pollution or more severe damage to the ecological environment. The utilization of corn stover resources can not only reduce the dependence on fossil energy but also has important practical and strategic significance for the development of a low-carbon economy [2].

The cell wall of lignocellulose mainly contains cellulose, hemicellulose, and lignin. In the process of converting lignocellulosic biomass (such as corn stover), it is necessary to pretreat it to reduce its resistance, allowing for the subsequent enzymolysis and fermentation processes. Dilute sulfuric acid pretreatment is among the most frequently used and industrially feasible pretreatment methods. However, various decomposition byproducts (such as furfural, phenols, and organic acids, etc.) are released into the prehydrolysate after dilute acid pretreatment, which will seriously inhibit the subsequent enzymatic hydrolysis reaction [3]. The backbone of the hemicellulose component in the biomass is largely acetylated, and the acetyl group content depends on the plant species and accounts for about l~6 wt% of the biomass [4]. The resistance of biomass to enzymatic digestion is known to be greatly influenced by acetyl groups [5]. The acetic acid in the prehydrolysate is formed by splitting covalently bound groups of acetyls in the principal chain of xylan during dilute acid pretreatment. The concentration of acetic acid in the pretreatment solution is much higher than that of any other inhibitors, and it has a significant impact on enzymatic saccharification [6]. Acetic acid at 1 g/L has the potential to inhibit cellulase activity by 10%, and its inhibitory effect increases significantly at higher levels [6]. Chen et al. demonstrated that 70% of the acetyl group removed after pretreatment will increase the enzymatic yield significantly [7]. Additionally, acetic acid affects the fermentation process and interferes with microbial growth. This biomass degradation product can lead to slower fermentation rates and lower yields, and the acidification of the cytoplasm inhibits the activity of enzymes required for microbial growth [8]. Wang et al. used a dilute alkaline deacetylation process combined with thermochemistry and mechanical pretreatment of corn stover and bagasse to increase the final glucose/xylose produced by enzymatic hydrolysis and ethanol yield by 6.5% [9].

There has been a lot of research on the detoxification of prehydrolysate to improve the bioconversion capacity of lignocellulosic raw materials, but the detoxification processes will bring a considerable increase in the cost and complexity of the production process. Common methods for detoxification, such as the addition of excessive amounts of lime, ion exchange, or activated carbon treatment, can lower the concentration of acids, furans, and phenolic compounds, thereby enhancing the efficiency of bioconversion [10]. However, some of these techniques require the use of costly bioprotein, which may not be economically feasible for the production process [11]. Methods with lower costs, like applying excessive amounts of lime and activated carbon adsorption, have certain limitations in their effectiveness and are more effective when used in combination with other detoxification methods [12]. Moreover, commonly used detoxification methods have low efficiency in removing organic acids, and the concentration of acetic acid is not significantly reduced [13]. Guo et al. investigated the removal efficiency of five potential inhibitors in spruce hydrolysate using activated carbon, ion exchange resins, and different alkalis. The results revealed that these detoxification methods achieved less than 40% removal rate for acetic acid while demonstrating good detoxification effects on other heterogeneity degradation products such as furfural and phenolics [14]. If the acetyl group can be removed from the substrates before dilute acid pretreatment, the production of the inhibitors can be greatly reduced, which is important for improving the subsequent enzymatic digestion and fermentation. Studies have shown that alkaline treatment is effective in removing acetyl groups from natural corn stover [15]. In general, alkaline deacetylation can be carried out under mild conditions, and the reaction can be completed at room temperature and pressure. But most of the bases used in the existing studies are strong bases like sodium hydroxide with a higher temperature, long reaction time, and hemicellulose degradation during the process [16]. In this study, Na_2_CO_3_ was selected as an inexpensive and greener deacetylation reagent for corn stover pretreatment under mild conditions.

This study explored the effect of sodium carbonate deacetylation of corn stover followed by dilute acid treatment, expecting to effectively remove acetyl groups from corn stover and improve the subsequent enzymatic hydrolysis.

## 2. Materials and Methods

### 2.1. Substrate and Chemicals

The corn stover was harvested from Shang Zhuang Experimental Station of China Agricultural University in Beijing, China. The corn stover was air-dried to a moisture content below 10% and then ground to the size of 20–60 mesh. The sieved samples were collected and sealed in bags and stored at room temperature for further use. The composition of corn stover was determined to be 32.83% cellulose, 22.19% hemicellulose, 19.93% lignin, 2.47% acetyl, and 4.96% ash according to NREL’s protocol [17]. Cellulase enzyme (Novozymes, Ctec-2) was purchased from Sigma-Aldrich (Shanghai, China) with an activity of 116 FPU/mL. All other chemicals were purchased from Beijing Chemical Works (Beijing, China).

### 2.2. The Deacetylation of Corn Stover with Sodium Carbonate

The sodium carbonate deacetylation conditions were as follows: the solid-to-liquid ratio was 1:15; the sodium carbonate solutions were in the range of 2~6 wt%; the temperature range was 30~50 °C; and the reaction times were between roughly 3 and 9 h. The reaction was carried out in flasks, and the temperature was maintained by a shaker incubator at 150 rpm. After the deacetylation was carried out, the solid was collected by vacuum separation with a Brinell funnel. The liquid was collected to determine sugar loss. The filtrated solids were repeatedly rinsed to neutral with deionized water, and then the solids were oven-dried for subsequent composition analysis and dilute acid pretreatment. The deacetylated samples were named D samples. The sample that has not undergone any treatment was named UT. All deacetylation experiments were carried out in duplicates. Hemicellulose loss and acetyl removal were calculated according to Equations (1) and (2). The solid recovery rate was calculated using Equation (3).

(1) $$\begin{array}{c} Hemicellulose\;loss\;\left(\%\right)\;=\;1\;-\;\frac{hemicellulose\;content\;in\;pretreated\;corn\;stover\;\times \;solid\;recovery}{hemicellulose\;content\;in\;raw\;corn\;stover} \end{array}$$

(2) $$\begin{array}{c} Acetyl\;removal\;\left(\%\right)\;=\;1\;-\;\frac{acetyl\;content\;in\;pretreated\;corn\;stover\;\times \;solid\;recovery}{acetyl\;content\;in\;raw\;corn\;stover\;} \end{array}$$

(3) $$\begin{array}{c} Solid\;recovery\;\left(\%\right)\;=\;\frac{Dry\;weight\;of\;the\;deacetylated\;solid\;after\;washing}{Dry\;weight\;of\;the\;initial\;solid\;} \end{array}$$

### 2.3. Dilute Acid Treatment of Deacetylated Corn Stover

The deacetylated corn stover was subjected to dilute acid treatment to break down its recalcitrance. The condition of dilute sulfuric acid pretreatment was as follows: dilute sulfuric acid with the mass fraction of 1.5%, 121 °C, 1 h, and the solid/liquid ratio of 1:15 [18]. Dilute sulfuric acid treatment was performed in high-pressure glass tubes in an autoclave (MLS-3750, Sanyo, Osaka, Japan). The solid–liquid separation after acid treatment was the same as the deacetylation step. The residual solid was collected after drying, and the sample loss was recorded. Dilute acid pretreatment of raw corn stover was also performed for comparison purposes. The solid residue was used for subsequent composition analysis and enzymolysis experiments. All dilute acid treatment experiments were carried out in duplicates.

### 2.4. Enzymatic Hydrolysis of Treated Corn Stover

Enzymatic hydrolysis was performed in a conical flask with the addition of 2% solids. The enzyme loading was 15 FPU/g substrate (20 mg protein/g substrate). The enzymatic hydrolysis substrates include untreated samples (UT), samples with only deacetylation (D), samples with only dilute acid pretreatment (DA), and samples with both deacetylation and dilute acid pretreatment (DDA). Whole slurry enzymatic hydrolysis involves the enzymatic hydrolysis of solid samples (DA and DDA) that have undergone dilute sulfuric acid pretreatment, along with the pretreatment solution generated during the process. The conditions of enzymatic hydrolysis were the same as in our previous reports [19]. The reaction was carried out at 150 rpm and 50 °C for 72 h. Samples were taken at 12 h intervals, and the supernatant, after centrifugation, was passed through a filter membrane into a chromatography bottle after inactivation and pH adjustment. The concentrations of glucose, xylose, and cellobiose in the samples were determined. All enzymatic hydrolysis experiments were carried out in duplicates. The enzymatic hydrolysis yield of glucose and xylose was calculated using the following equations:

(4) $$\begin{array}{c} Glucose\;yield\left(\%\right)\;=\;\frac{Glucose\;release\left(g\right)\;+\;1.053\;\times \;Cellobiose\;release(g)}{1.111\;\times \;Glucan\;content(g)} \end{array}$$

(5) $$\begin{array}{c} Xylose\;yield\left(\%\right)\;=\;\frac{Xylose\;release\left(g\right)}{1.136\;\times \;xylan\;content(g)} \end{array}$$

### 2.5. Composition Analysis of Treated Samples

The determination of cellulose, hemicellulose, lignin, and ash content in corn stover before and after deacetylation treatment was performed using the NREL’s standard method (NREL-TP-510-42618 to 42622) [17]. The oligomers were quantified according to NREL-TP-510-42623 [17]. The sugars in the hydrolysates were measured by HPLC. Aminex HPX-87P column (Bio-Rad, Hercules, CA, USA) was applied for the analysis at 80 °C, and ultrapure water was used as the mobile phase at a flow rate of 0.6 mL/min.

### 2.6. Scanning Electron Microscopy (SEM) Analysis

The surface micromorphological changes of the samples before and after sodium carbonate deacetylation and dilute sulfuric acid pretreatment of corn stover were observed by a Japanese Hitachi SU3500 electron microscope. (Hitachi, Tokyo, Japan). The method was described in detail in our previous report [18].

### 2.7. FT-IR Analysis

Changes in functional groups (mainly acetyl groups) in corn stover samples before and after different treatments were measured qualitatively using a Fourier transform infrared spectrometer (Spectrum 400; PerkinElmer, Waltham, MA, USA). The method is the same as our previous report [18]. Each sample was measured twice.

### 2.8. Statistical Analysis

All statistical analyses were performed with the SPSS 26.0 statistical package (IBM, Armonk, NY, USA). Values are presented as the mean ± standard deviation for data that were normally distributed in all comparisons. 

## 3. Results and Discussion

### 3.1. The Compositional Change of Corn Stover after Sodium Carbonate Deacetylation

The solid fraction of corn stover before and after deacetylation was analyzed, and their composition was summarized in Table 1. Different from traditional alkali treatment methods, which mainly aim to remove lignin, alkali deacetylation primarily focuses on selectively removing acetyl groups under milder conditions while maximizing the retention of fermentable sugars. During deacetylation, the mass loss of corn stover ranged from about 18% to 25%. Compared to other alkaline treatment methods using sodium hydroxide, Goshadrou et al. reported a solid recovery rate of 66% [20], while Prajapati et al. reported a solid recovery rate of 40% [21]; both results showed that the solid recovery was significantly improved. Under different deacetylation conditions, the cellulose maintained a high recovery rate, and the increase of cellulose content after pretreatment could obtain higher glucose concentration in the subsequent enzymatic hydrolysis step. However, the removal of hemicellulose and lignin varied greatly under different pretreatment conditions. Alkaline treatment has a good delignification effect on biomass; the lignin content of the deacetylated corn stover was lower than that of the untreated corn stover.

### 3.2. Effect of Temperature on Na_2_CO_3_ Deacetylation of Corn Stover

**Figure 1:**
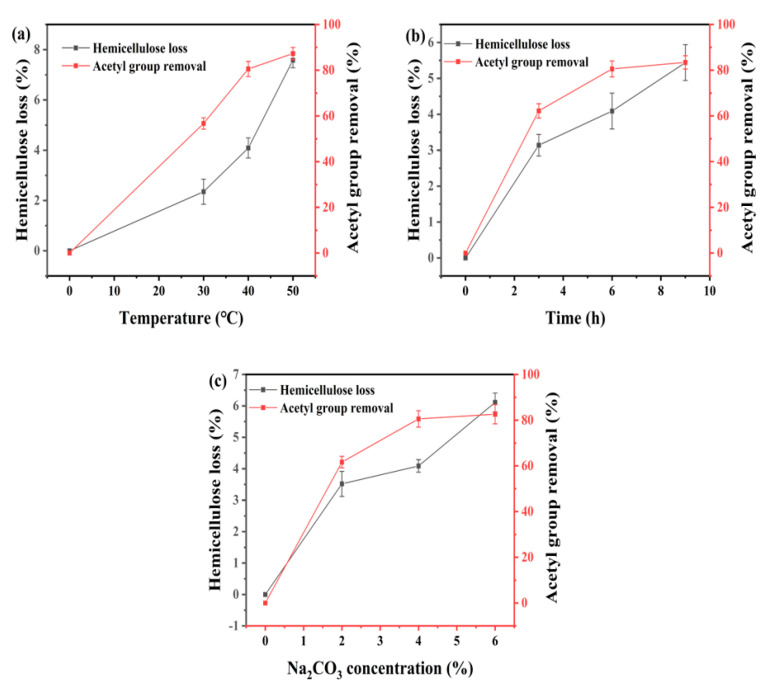
The impact of different factors on the deacetylation efficiency of sodium carbonate. (**a**) Temperature effect. Deacetylation conditions: 4% Na_2_CO_3_, 6 h. (**b**) Treatment time effect. Deacetylation conditions: 4% Na_2_CO_3_, 40 °C. (**c**) Na_2_CO_3_ concentrate effect. Deacetylation conditions: 6 h, 40 °C.

Three temperatures (30 °C, 40 °C, and 50 °C) were used to investigate the effect of temperature on the deacetylation of corn stover. The concentration of Na_2_CO_3_ and the treatment time were kept the same at 4% and 6 h, respectively, for all three temperatures. As shown in Figure 1a, the temperature showed a more significant impact on the hemicellulose loss than the acetyl removal because hemicellulose is more sensitive to chemical attack among the three major components of lignocellulosic biomass [22]. At 30 °C, 2.35% hemicellulose degradation was observed with an acetyl group removal rate of 56.70%. Increasing the temperature to 40 °C resulted in a hemicellulose loss of 4.09% and an acetyl removal rate of 80.55%. For Na_2_CO_3_ deacetylation at 50 °C, the acetyl removal was 87.24%, which was only 7% higher than that at 40 °C. Meanwhile, the hemicellulose loss at 50 °C almost doubled to 7.58%. The loss of hemicellulose increased more rapidly with the rise of temperature during alkaline treatment, which indicates that higher temperatures are not favorable for deacetylation in terms of hemicellulose preservation. Among the three temperatures studied, 40 °C would be an optimal choice with satisfactory acetyl removal and low hemicellulose loss.

### 3.3. Effect of Reaction Time on Deacetylation of Corn Stover

Three reaction times (3 h, 4 h, and 5 h) were used to investigate the effect of reaction time on the deacetylation of corn stover. The concentration of Na_2_CO_3_ and the treatment temperature were kept the same at 4% and 40 °C, respectively, for all three treatment times. The effect of reaction time on deacetylation is mainly reflected in the fact that increasing reaction time can improve the removal of the acetyl group but also reduce the selectivity of the reaction (increased hemicellulose loss). As shown in Figure 1b, when the deacetylation time was 3 h, the acetyl content in corn stover decreased from 2.47% to 0.85%, with an acetyl removing rate of 62.21% and the hemicellulose loss of 3.16%. At 6 h, 4.09% hemicellulose degradation was observed with an acetyl group removal rate of 80.55%. When the pretreatment time was increased to 9 h, the hemicellulose loss was 5.44%, while the acetyl removal only increased from 80.55% to 83.43%. The loss of hemicellulose and the degree of deacetylation of biomass increased with the increase of deacetylation time. However, when the deacetylation time was more than 6 h, the prolonged reaction time had little effect on the removal of acetyl groups. Therefore, 6 h would be a better deacetylation time with satisfactory acetyl removal and acceptable hemicellulose loss. The study by Shekiro et al. also showed that the reaction time is only a minor factor in the deacetylation process, and the reaction is largely driven by the reaction temperature and alkaline load [23].

### 3.4. Effect of Na_2_CO_3_ Concentration on Deacetylation of Corn Stover

Three Na_2_CO_3_ concentrations (2%, 4%, and 6%) were used to investigate the effect of Na_2_CO_3_ concentration on the deacetylation of corn stover. The reaction time and the treatment temperature were kept the same at 6 h and 40 °C, respectively, for all three treatment times. Figure 1c shows the effect of different mass fractions of Na_2_CO_3_ solution on deacetylation. When the mass fraction of sodium carbonate was 2%, hemicellulose degradation of 3.52% was observed, with an acetyl group removal rate of 61.66%. When the Na_2_CO_3_ concentration was increased to 4%, the hemicellulose loss was 4.09%, and the acetyl removal was raised to 80.55%. For Na_2_CO_3_ deacetylation at 6%, the acetyl removal was 82.61%, and the hemicellulose loss was 6.11%. Therefore, when the solid–liquid ratio and other conditions are consistent, the degree of deacetylation will be positively correlated with the mass fraction of the base [24]. In this experiment, it can also be observed that the degree of deacetylation increased with the increase of base load, but there is no significant correlation between the two, probably due to the low temperature selected in the experiment and the weak alkaline used. A sodium carbonate concentration of 4% would be an optimal level for deacetylation of corn stover.

According to the deacetylation parameter analysis, the optimal condition was determined to be 40 °C, 6 h, and 4% Na_2_CO_3_ mass fraction. The hemicellulose loss at this condition was 4.09%, and the acetyl group removal rate was 80.55%. Zhang et al. reported the deacetylation reaction of sugarcane bagasse under the conditions of 0.1 M NaOH at 80 °C for 3 h. The acetyl group removal rate was found to be 70%. However, simultaneously, 19.2% of glucan and 31.9% of xylan were also removed [9]. The loss of sugar occurs during the deacetylation process, while sodium carbonate can effectively remove acetyl groups while preserving carbohydrates in biomass.

### 3.5. Effect of Sodium Carbonate Deacetylation on Dilute Acid Pretreatment

The deacetylated corn stover was subjected to dilute acid treatment to break down its recalcitrance. Dilute acid pretreatment of raw corn stover was also performed for comparison purposes. The pretreated samples with only dilute sulfuric acid were named DA samples, and the samples with deacetylation and dilute sulfuric acid treatment were named DDA samples. Table 2 shows the solid composition after the dilute sulfuric acid pretreatment and the concentration of acetic acid in the acid prehydrolysate. After pretreatment with dilute acid, the mass recovery of deacetylated samples was between 64.6% and 70.4%. The mass loss of 30~40% was mostly hemicellulose dissolved in the dilute acid pretreatment process. The removal of hemicellulose was crucial for subsequent enzymatic hydrolysis and fermentation because it increases the accessibility of cellulose to enzymes, which facilitates the enzymatic breakdown process [25]. There were no significant differences in the cellulose, hemicellulose, and lignin content in the deacetylated samples after dilute sulfuric acid pretreatment.

**Figure 2:**
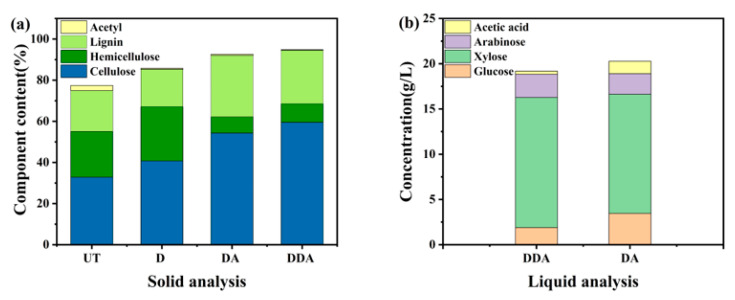
(**a**) Composition analysis of corn stover samples from different pretreatment. (**b**) Liquid analysis after dilute sulfuric acid pretreatment; UT: untreated corn stover; D: deacetylated corn stover (4% Na_2_CO_3_, 40 °C, 6 h); DA: dilute-acid-treated corn stover (1.5% H_2_SO_4_, 121 °C, 1 h); DDA: D + DA treated corn stover.

The differences in solid sample composition under different treatment conditions were analyzed and compared in Figure 2a. The difference in the content of solid samples was mainly reflected in the hemicellulose and acetyl groups because the two-step pretreatment process selectively removed the acetyl group and part of the hemicellulose. In addition, it was also seen that the lignin content decreased after two-step pretreatment, which may have an impact on the subsequent enzymatic process. Figure 2b shows the composition of the prehydrolysate of the DDA sample, and it was compared with that of the DA sample. The sugars in the prehydrolysate were mainly hemicellulose sugars. The acetic acid concentration of the raw corn stover sample was 1.38 g/L in the prehydrolysate, and the concentration of acetic acid was significantly reduced after deacetylation. The lowest acetic acid concentration was 0.14 g/L. For the sample generated at the optimal deacetylation condition, the acetic acid concentration in the prehydrolysate was 0.34 g/L, which is 75.72% lower than the raw sample. The content of other decomposition products, such as furfural and hydroxymethyl furfural in the prehydrolysate, was very low.

### 3.6. Effect of Na_2_CO_3_ Deacetylation on Enzymatic Hydrolysis

**Figure 3:**
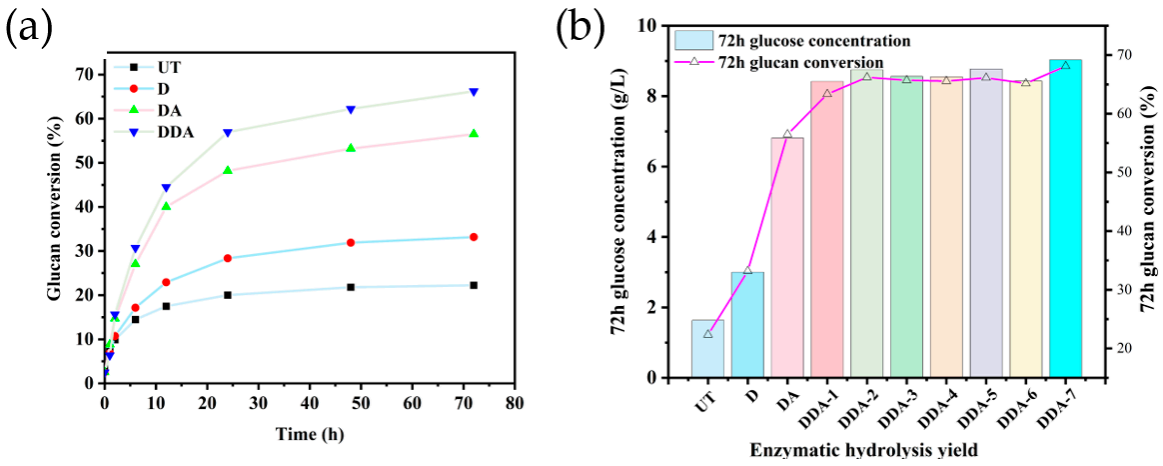
(**a**) Curve of the conversion rate of glucan over time. (**b**) Enzymatic hydrolysis performance of corn stover solids treated under different conditions. Enzymatic hydrolysis condition: 72 h, 2% solid loading, 20 mg protein/g substrate.

To investigate the effect of sodium carbonate deacetylation on enzymatic hydrolysis of corn stover, the solid fraction from the two-step treatment (deacetylation + dilute sulfuric acid pretreatment, which was marked as DDA samples), dilute acid pretreatment alone (which was marked as DA samples), as well as deacetylation treatment alone (which was marked as D samples) were subjected to enzymatic digestion tests at 2% solid loading; the cellulase enzyme loading was 15 FPU/g substrate. The time-progressing curve of glucose conversion and the final glucose concentration for different samples are shown in Figure 3a and Figure 3b, respectively. The conversion of glucan for both the D and the DA samples was higher than that of the untreated sample (UT). For corn stover with a high hemicellulose content, the acetyl group on hemicellulose is the main resistance obstacle because the acetyl spatial blockage reduces enzyme activity. Therefore, the hydrolysis yield of glucan can be improved by selectively removing the acetyl group from the xylan skeleton of raw materials through deacetylation. Selig et al. showed that the acetyl group bound to the main chain of xylan hindered the hydrolysis of cellulase to β-1,4 glucoside bonds, while the deacetylation of enzymes provided more binding sites [26]. But due to the mild deacetylation condition applied in this study, the highest glucan conversion was only 33.18% from deacetylation alone treatment compared to 22.24% from the UT group. The alkaline deacetylation treatment in this study was used as a supplementary step to remove the acetyl groups while preserving the carbohydrates in corn stover before dilute acid pretreatment. It was not aimed to obtain a high yield from deacetylation alone. With the help of deacetylation, the glucan digestibility of the DDA sample showed clear improvement. Compared to the DA sample with a glucose yield of 56.48%, the glucan conversion of the DDA sample increased to 66.21%. Júlia Ribeiro Martins et al. employed dilute sulfuric acid pretreatment on sugarcane leaves (2% H_2_SO_4_, 120 °C, 1 h), resulting in a cellulose enzymatic conversion rate of 69.84% [27]. Due to the lower acid concentration used in our experiment (only 0.5%), the enzymatic conversion rate was lower with only dilute acid pretreatment. However, after alkaline deacetylation treatment, there was a significant improvement in the enzymatic conversion rate. Additionally, there are studies indicating that the presence of acetyl groups can potentially modify the hydrophilicity of cellulose, thereby affecting the binding of cellulose and cellulase enzymes through hydrogen bonding [28]. Low solid concentration during enzymatic hydrolysis typically exhibits higher conversion rates [29]. However, under high solid conditions, the enzymatic conversion rate decreases as the solid loading increases due to factors such as adsorption, diffusion, and inhibition of enzyme activity by product concentration [30]. Under high solid process conditions, deacetylation can significantly reduce the concentration of inhibitory compounds in the enzymatic hydrolysis solution, making the role of alkali deacetylation more prominent.

**Figure 4:**
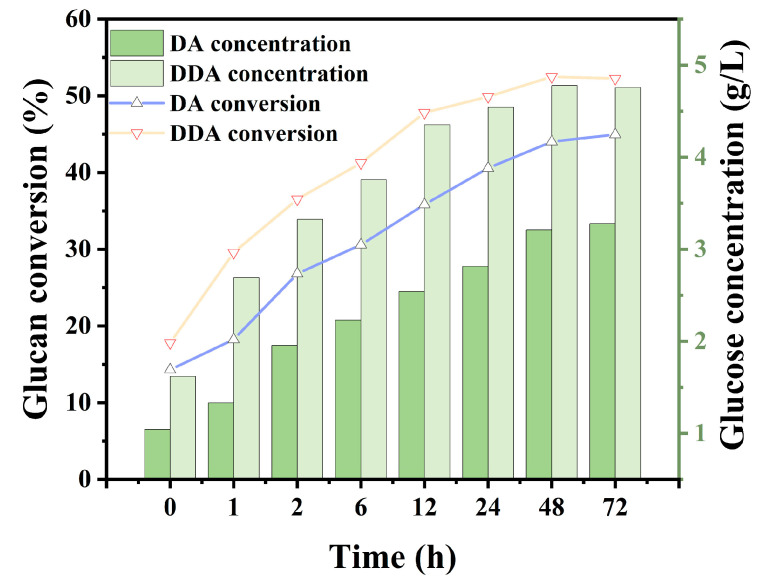
Whole slurry enzymatic hydrolysis of acid-treated corn stover and deacetylated + dilute-acid-treated corn stover. Enzymatic hydrolysis condition: 72 h, 2% solid loading, 20 mg protein/g substrate.

As shown in Table 2 and Figure 2b, the major effect of deacetylation was the reduced acetic acid concentration in the prehydrolysate after the dilute acid treatment. The ultimate purpose of deacetylation was to achieve the total utilization of both the solid and liquid stream from biomass pretreatment. The prehydrolysate contains a substantial amount of fermentable sugar that must be utilized. To evaluate the effectiveness of the deacetylation process, the whole slurry from the DDA and DA treatment was subjected to enzymatic hydrolysis without solid–liquid separation. The pH of the slurry was adjusted to 4.8 before enzymatic hydrolysis. No further detoxification process was applied. The enzymatic hydrolysis process for whole slurry was similar to that of solid enzymatic hydrolysis. The results of the whole slurry hydrolysis experiment are shown in Figure 4. Due to the presence of degradation compounds in the prehydrolysate, a decrease in glucose yield was observed in the whole slurry enzymatic hydrolysis. Jiang et al. have shown that high concentrations of acetyl groups can partially or completely inhibit the enzymatic hydrolysis process [31]. The glucan conversion rates of the DA and the DDA sample were 44.95% and 52.25%, respectively. The 16% increase in the DDA sample could be attributed to the lower acetic acid concentration in the prehydrolysate, which resulted from the alkaline deacetylation. No discernable changes in other presentative inhibitors, such as furfural and HMF, were noticed between the DA and DDA samples.

### 3.7. SEM Analysis and FTIR Analysis

**Figure 5:**
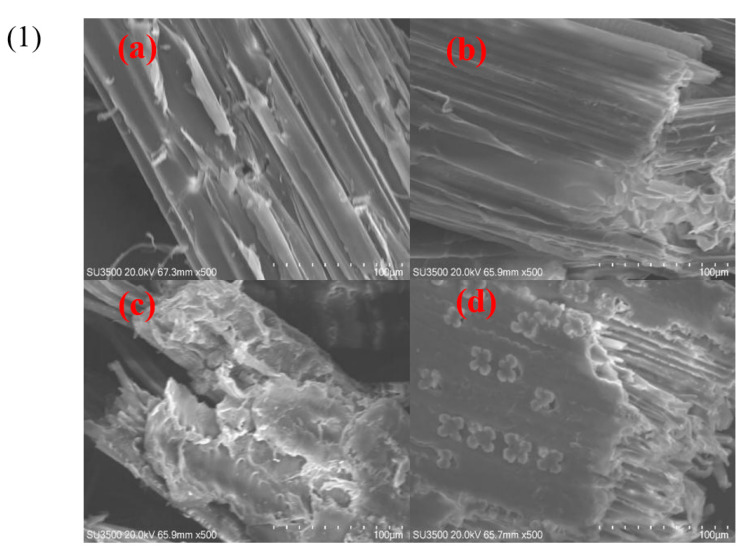

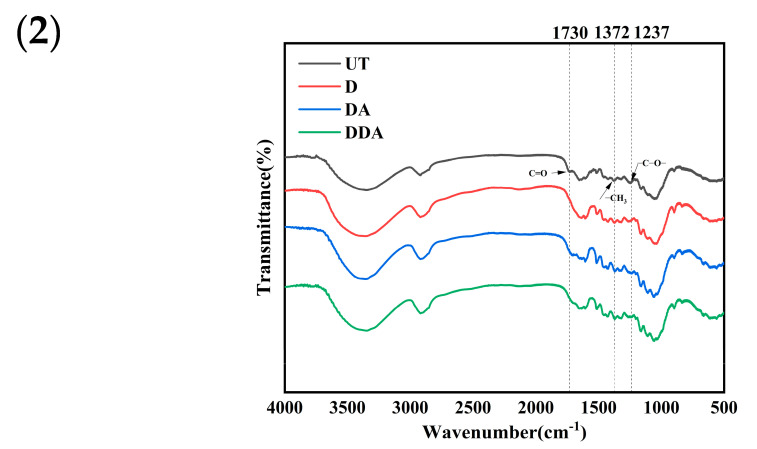
(**1**) SEM analysis of substrates. (**a**) UT; (**b**) D; (**c**) DA; and (**d**) DDA. (**2**) FT-IR analysis of substrates.

The structural changes of untreated and treated corn stover were imaged by SEM (Figure 5(1)). The surface appearance of the untreated corn stover (Figure 5(1a)) had a more regular surface structure with layers of lignin, hemicellulose, and cellulose wrapped from the outside to the inside. The dense structure makes the raw biomass difficult for the enzyme to effectively contact and hydrolyze cellulose. After deacetylation (Figure 5(1b)) with sodium carbonate, the surface morphology of the corn stover became smoother due to the removal of a small amount of hemicellulose and lignin, but it still did not break its stubborn structure. After pretreatment with dilute sulfuric acid, the particle size appeared to be smaller, along with noticeable holes and cracks (Figure 5(1c)). The cell structure was broken, and part of the hemicellulose was removed, leading to the exposure of cellulose and providing more binding sites for cellulase. These changes facilitated the rapid and efficient hydrolysis of cellulose. Unfortunately, no clear differences were observed for the two-step treated corn stover, as shown in (Figure 5(1d)). FTIR was used to observe changes in the main functional groups of corn stover during alkaline deacetylation and dilute sulfuric acid pretreatment (Figure 5(2)). The characteristic peaks for acetyl groups were found to be near 1730 cm^−1^, 1372 cm^−1^, and 1237 cm^−1^ delegated for C=O, –CH_3_, and –C–O–, respectively [32,33]. Compared to raw corn stover without any treatment, the absorption peak strength of D, DA, and DDA samples was significantly reduced, which was consistent with the results of the chemical composition analysis. Moreover, the intensity of the absorption peak was positively correlated with the removal rate of the acetyl group. In addition, the analysis of the characteristic peaks of 1214 cm^−1^, 1423 cm^−1^, and 1740 cm^−1^ showed that the chemical structures of the three main components in corn stover did not undergo substantial changes during the deacetylation process.

## 4. Conclusions

Alkaline deacetylation of corn stover at 40 °C using 4 wt% Na_2_CO_3_ could effectively remove more than 80% of the acetyl of corn stover while maintaining the xylan loss at around 4%. The acetic acid concentration in the acid prehydrolysate can thus be reduced by more than 75%. The enzymatic hydrolysis performance on the solid corn stover after the DDA treatment was 17% higher than that from the DA treatment. For the whole slurry hydrolysis, the glucan conversion rates of the DA and the DDA sample were 44.95% and 52.25%, respectively. The 16% increase in the DDA sample could be attributed to the lower acetic acid concentration in the prehydrolysate, a result of the alkaline deacetylation. Alkaline deacetylation can improve the corn stover enzymatic performance after dilute acid pretreatment. Sodium carbonate proved to be an effective deacetylation agent with the advantages of being cost-effective and environmentally friendly compared to commonly used strong base agents like sodium hydroxide. In the future, exploration in recovering and reusing the sodium carbonate from the deacetylation of black liquor through the Kraft pulping process could be studied further, which would enhance the overall economic feasibility of the entire process.

## Figures and Tables

**Table 1:** Composition analysis of raw and deacetylated corn stover.

Deacetylation Condition			Cellulose	Cellulose	Hemicellulose	Hemicellulose	Lignin	Lignin	Acetyl	Acetyl	Solid
Temp	Time	Na_2_CO_3_	(%)	Loss (%)	(%)	Loss (%)	(%)	Remove (%)	(%)	Remove (%)	Recovery (%)
Raw (control)			32.83 ± 0.23	-	22.19 ± 0.31	-	19.93 ± 0.62	-	2.47 ± 0.23	-	-
40 °C	6 h	2%	40.85 ± 0.11	0.70 ± 0.14	26.40 ± 0.28	3.52 ± 0.39	18.38 ± 0.51	26.40 ± 0.83	1.13 ± 0.11	61.66 ± 2.48	79.80 ± 0.92
40 °C	6 h	4%	40.69 ± 0.16	1.62 ± 0.23	26.39 ± 0.36	4.09 ± 0.35	18.21 ± 0.37	27.47 ± 0.42	0.44 ± 0.04	80.55 ± 3.36	79.38 ± 1.23
40 °C	6 h	6%	40.92 ± 0.34	2.38 ± 0.28	26.18 ± 0.52	6.11 ± 0.32	18.29 ± 0.42	28.15 ± 0.66	0.40 ± 0.02	82.61 ± 4.23	78.31 ± 0.80
40 °C	3 h	4%	40.45 ± 0.27	1.40 ± 0.31	26.44 ± 0.13	3.14 ± 0.28	19.21 ± 0.36	22.89 ± 0.71	0.85 ± 0.05	62.21 ± 3.12	80.02 ± 1.35
40 °C	9 h	4%	41.32 ± 0.42	2.24 ± 0.37	26.59 ± 0.60	5.44 ± 0.47	18.22 ± 0.64	28.97 ± 0.41	0.39 ± 0.02	83.43 ± 2.95	77.68 ± 0.69
30 °C	6 h	4%	39.53 ± 0.26	1.60 ± 0.29	26.10 ± 0.44	2.35 ± 0.51	19.26 ± 0.58	21.04 ± 0.67	0.96 ± 0.04	56.70 ± 2.50	81.72 ± 1.04
50 °C	6 h	4%	42.91 ± 0.50	2.36 ± 0.33	27.02 ± 0.67	7.58 ± 0.31	18.02 ± 0.71	32.48 ± 0.92	0.31 ± 0.06	87.24 ± 2.71	74.70 ± 1.62

**Table 2:** Composition of corn stover after 2-step treatment and the acetic acid concentration in dilute acid prehydrolysate.

Sample	Deacetylation Condition			Dilute Sulfuric Acid Pretreatment	Cellulose	Hemicellulose	Lignin	Acetic Acid
	Temp	Time	Na_2_CO_3_		(%)	(%)	(%)	Concentrate (g/L)
Raw				1.5 wt% H_2_SO_4_, 121 °C, 1 h				
DDA-1	40 °C	6 h	2%		59.88 ± 0.26	9.01 ± 0.25	26.03 ± 0.60	0.77 ± 0.08
DDA-2	40 °C	6 h	4%		59.50 ± 0.43	9.28 ± 0.22	25.62 ± 0.50	0.34 ± 0.04
DDA-3	40 °C	6 h	6%		58.60 ± 0.15	9.41 ± 0.34	26.04 ± 0.28	0.21 ± 0.03
DDA-4	40 °C	3 h	4%		58.69 ± 0.36	8.91 ± 0.20	26.48 ± 1.02	0.50 ± 0.05
DDA-5	40 °C	9 h	4%		60.21 ± 0.28	9.74 ± 0.14	24.68 ± 0.79	0.17 ± 0.03
DDA-6	30 °C	6 h	4%		58.25 ± 0.42	8.75 ± 0.11	27.11 ± 0.43	0.67 ± 0.05
DDA-7	50 °C	6 h	4%		59.67 ± 0.23	9.80 ± 0.30	24.22 ± 0.56	0.14 ± 0.02

## Data Availability

No new data were created or analyzed in this study. Data sharing is not applicable to this article.
